# Emergence of Omicron BA.1 and BA.2 variants and concern over vaccine breakthrough infection

**DOI:** 10.1016/j.amsu.2022.103941

**Published:** 2022-06-06

**Authors:** Mahnoor Sukaina, Mohammad Mehedi Hasan, Mohammad Yasir Essar

**Affiliations:** Karachi Medical and Dental College, Karachi, Pakistan; Department of Biochemistry and Molecular Biology, Faculty of Life Science, Mawlana Bhashani Science and Technology University, Tangail, Bangladesh; Afghanistan National Charity Organization for Special Diseases (ANCOSD), Kabul, Afghanistan; Kabul University of Medical Sciences, Kabul, Afghanistan

**Keywords:** Vaccine breakthrough infection, COVID-19, BA.1 variant, BA.2 variant, SARS-CoV-2, Recommendation third booster dose, Fourth recommended COVID-19 vaccine

## Abstract

The COVID-19 pandemic since 2020 created havoc across the globe. This led to the establishment of effective vaccines to mitigate the virus. Several vaccines are developed to create immunity amongst the population. However, due to emerging variants of SARS-CoV-2, despite a double dose of vaccines, people have still infected that termed vaccine breakthrough infection. This paper aims to highlight the recent re-surging massive outbreak of COVID-19 in China despite mass vaccination and, discuss the issue of vaccine breakthrough infection, possible causes, and recommendations to enhance immunity and curb the transmission.

## 1 Resurgence of massive COVID-19 outbreak in China

The incessant COVID-19 pandemic has shaken the core of health infrastructure across the world. According to the World Health Organization (WHO), as of 11^th^ March 2022, more than three billion doses of the COVID-19 vaccine have been administered in China [1]. However, despite mass vaccination across the nation, China has reported a massive outbreak of SARS-CoV-2 since the start of the pandemic in the two years. According to the National Health Commission of China, as of March 19th^,^ 2022, 1737 new COVID-19 cases are reported, with a total of 130,199 confirmed cases and more than four thousand total deaths [2]. The cases are not only limited to China but the transmission of the deadly virus is spreading to neighboring countries at an exponential rate. Recently, Hong Kong reported 275,783 confirmed COVID-19 cases [2]. Nevertheless, not as a vaccine breakthrough consequence but, Hong Kong has failed to curb the transmissibility of the infection that accounts for 40%, however, 90% of the people dying due to the COVID-19 are unvaccinated [3].

## 2 Vaccine breakthrough infection and high infectivity and transmission rate

Amidst the BA.1 omicron transmission causing vaccine breakthrough infection globally, a superimposed “stealth” BA.2 has emerged to cause a catastrophic escalation in COVID-19 cases in China. The BA.2 is responsible for more infectivity and a greater transmission rate than the previous BA.1 omicron variant [4]. COVID-19 vaccines are effective at preventing serious illness and death. A vaccine breakthrough infection is the detection of SARS- CoV-2 RNA or an antigen in a respiratory tract collected from a person ≥14 days after the reception of all recommended doses of an FDA-authorized COVID-19 vaccine [5]. FDA-authorized vaccines are effective, but there are chances of being infected until immunity reaches a sufficient level to further decrease transmission. It is important to know that coronavirus encompasses not only spike protein but also includes membrane, envelope, hemagglutinin-esterase, and nucleocapsid proteins [6]. Early-stage research has found that the other four proteins in the virus can exhibit or inhibit the viral mutations [9], and could be accountable for breakthrough infections. Delta variant that was first identified in India, rapidly transmitted to the rest of the world, made a parabolic increase to 83% of the new infections in the US in July 2021, which was only 10% at the start of June 2021 [7]. The reason behind the higher virulence and transmissibility of the Delta variant is its BFE and higher antibody-RBD complexes disruption [7.8]. Currently, it is the beta variant that has the highest potential to break through the vaccines, however, due to its lower infectivity, it is not deemed as posing a grave threat. However, in the future, the Lambda co-mutant variant has the potential to cause the vaccine breakthrough infection due to its second-highest BFE after Delta, and greater antibody disruptions than the Delta [8,9]. The RSYLTPGD246-253 N; a unique 7-amino-acid deletion mutation in the N-terminal domain of the Lambda S protein is primarily responsible for the escape from neutralizing antibodies [7,9]. It is concluded by a study that antibody neutralization is more likely to be present in, previous breakthrough infections in vaccinated individuals. In contrast to unexposed to the vaccine, breakthrough infections are highly susceptible to developing symptoms because the omicron variant is least likely to neutralize by antibodies. In addition to that, an interval between vaccination and breakthrough infection decides the fate of potency of neutralization in vaccines [10]. However, in contrast to spike gene deletion in BA.1 variant, the BA.2 sub-variants do not induce spike gene deletion at 69–70 positions. Subsequently, elucidates the S-gene target positive on the diagnostic test and shows more than 99% cases of S-gene target positive [11]. Thus, the new “stealth” BA.2 variant induces more infectivity and a greater transmission rate also with exposed household ornaments [11].

According to Dr. Maria Van Kerkhove, a World Health Organization (WHO) podcast, reported a surge in Omicron variant detection in more than 77 countries. She added, it is critical that even if we do see more mild diseases, everything is still to be done to decrease transmission in all populations vaccinated or not vaccinated [12]. Although the infection rate reported in vaccinated people is not as common as in unvaccinated. In another study, performed on two vaccinated patients, it was concluded that both patients’ immune systems responded effectively to the vaccine. Although it would have been ideal if a baseline antibody test before illness and after vaccination was conducted, it remains possible that she became infected before the booster shot took full effect [12]. There is a higher rate of Omicron detection in immunocompromised patients, therefore, they are recommended to follow all the safety protocols. In addition, CDC approves an additional dose of primary vaccine for immunocompromised patients [13]. Living in an area with a substantial or high transmission rate, following Standard Operating Procedure (SOP) is crucial even after being fully vaccinated.

Patients with vaccine breakthrough infections have reported less severe symptoms and low mortality, they are less likely to suffer any somber consequences but they are contagious. The COVID-19 vaccine mainly attacks the S protein, 32 amino acid changes, including three deletions and one insertion in the spike protein, demonstrate that Omicron may be induced by antibody resistance and results in more Omicron infections [14]. Vaccine breakthrough infections with Omicron raise serious concerns, as the Omicron variant has resulted in over 50 mutations along with 15 mutations in the Receptor Binding Domain (RBD). In a study, overall, it was illustrated that Omicron is expected to escape the vaccine-induced protection, perhaps a third homologous inactivated vaccine booster might be effective against Omicron [15]. Hospitalizations among vaccine breakthrough COVID-19 infections were found in patients who had received at least one dose of the vaccine or had not completed the full vaccine course. Vaccines have undoubtedly conferred widespread protection against SARS-CoV-2 infection, further research is needed to recognize and alleviate factors that are correlated with inadequate vaccine response in those with breakthrough infections [16]. Moreover, with the new BA.2 “stealth” sub-variant of omicron, it is controversial to assume breakthrough infection in BA.2 variants [17,18]. However, a recent study by Chen and Wei (2022), demonstrates that the BA.1, BA.2, and BA.3 pose the potential to evade vaccine protection and cause breakthrough infection. The looming cases of BA.2 which are superimposed on already vaccine breakthrough infection may create a drastic and detrimental response in health care infrastructure. Moreover, the BA.2 is 30% more potent than BA.1 and 17 times more than Delta variants to cause vaccine breakthrough infection [18].

## 3 Recommendations and conclusion

Following a systemic review of vaccine breakthrough infections by SARS-CoV-2 Delta Variant by Zhang et al. (2021), it is an area of concern that the vaccinated healthcare workers are being affected by the vaccine breakthrough Delta variant infections, so in this regard, more studies on targeted vaccines are needed. The research community postulates that most breakthrough infection patients were diagnosed with upper respiratory tract infections, hence these vaccines have little credibility over them. Studies on tissue-resident memory B cells in the respiratory mucosa should be done to increase the effect of these vaccines via humoral immunity [19]. Currently, to impede the combination between AXL and NTD which is associated with the entrance of virus into the host cells of vaccinated patients, a T cell-based adoptive cellular immunotherapy is being considered. Another study on monoclonal antibodies is done according to which CTP59, a monoclonal antibody, has shown better efficacy against Delta, Epsilon, and Kappa variant. Recently, antiviral drugs such as 3CL protein inhibitors and RNA synthesis inhibitors are also being considered as potential treatments for such infections [19]. These breakthrough infections are very common and severe in immunocompromised (IC) individuals even with active treatment status. According to CDC recommendations and FDA authorization, the 3rd dose of vaccine can maximize the protection of IC individuals against breakthrough infections. Hence, necessary measures should be taken for vaccine uptake among the IC [20]. Omicron transmission is much faster as compared to other variants and breakthrough infections are also expected, treatment with some, but not all, monoclonal antibodies have remained effective against Omicron. Delta variant also spreads easily, and breakthrough infections are common, but all FDA-authorized monoclonal antibody treatments show good efficacy against it [21]. In a most recent study by Kuhlmann et al. (2022), breakthrough infection by Omicron despite mRNA vaccine booster dose has been reported in a limited number of individuals, all these individuals had mild to moderate symptoms, so it suggests that the booster dose at least protects the severe form. Taking the given data into consideration, it is suggestive to promote updated vaccines to improve the protection against symptomatic infection with Omicron [22]. The study by Chang and Wei supports that sotrovimab (S309) is the sole mAb that does compete with RBD which is the main target of the omicron variant. Thus, escape the omicron breakthrough infection and preventive against SARS-CoV-2.

Due to the emerging variants of Omicron i.e. BA.1 and BA.2, in China, the zero-COVID policy is facing a major test as the daily virus figures have surged to a two-year high. Many researchers are recommending boosters to take up arms against the emerging variants. Messenger RNA (mRNA) vaccines should be considered for third, booster shots, instead of inactivated-virus vaccines that China has been previously using, as they might provide better immunity [23]. According to a recent study by Yochay R.G et al. (2022), the fourth dose of mRNA vaccine shows greater efficacy primarily against symptomatic disease. It suggests that mRNA vaccines offer maximal immunogenicity after three doses, but a fourth dose reinstates the antibody levels. However, in healthcare workers, low vaccine efficacy was noticed along with high viral loads advocating that the infected ones were infectious. As a consequence, the fourth shot of healthy young healthcare workers may have only marginal benefits [24].

## Ethical approval

N/A.

## Sources of funding

None.

## Registration of research studies

N/A.

## Consent

N/A.

## Guarantor

N/A.

## Provenance and peer review

Not commissioned, externally peer reviewed.

## Declaration of competing interest

None.
